# A national cohort study and confidential enquiry to investigate ethnic disparities in maternal mortality

**DOI:** 10.1016/j.eclinm.2021.101237

**Published:** 2021-12-13

**Authors:** Marian Knight, Kathryn Bunch, Nicola Vousden, Anita Banerjee, Philippa Cox, Fiona Cross-Sudworth, Mandish K. Dhanjal, Jenny Douglas, Joanna Girling, Sara Kenyon, Rohit Kotnis, Roshni Patel, Judy Shakespeare, Derek Tuffnell, Meg Wilkinson, Jennifer J. Kurinczuk, MBRRACE-UK

**Affiliations:** a Policy Research Unit in Maternal and Neonatal Health and Care, National Perinatal Epidemiology Unit, Nuffield Department of Population Health, University of Oxford, United Kingdom; b Guys and St Thomas’ Hospitals NHS Foundation Trust, London, United Kingdom; c Homerton University Hospital NHS Foundation Trust, London, United Kingdom; d Institute of Applied Health Research, University of Birmingham, United Kingdom; e Queen Charlotte's and Chelsea Hospital, Imperial College Healthcare NHS Trust, London, United Kingdom; f School of Health, Wellbeing and Social Care, Faculty of Wellbeing, Education and Language Studies, The Open University, United Kingdom; g Chelsea and Westminster Hospital NHS FT, London, United Kingdom; h General Practitioner, Oxford, United Kingdom; i Retired GP, Oxford, United Kingdom; j Bradford Hospitals NHS Foundation Trust, Bradford, United Kingdom; k University College London Hospitals, London, United Kingdom

**Keywords:** Maternal Mortality, Confidential Enquiry, Ethnic disparity

## Abstract

**Background:**

Ethnic disparities in maternal mortality were first documented in the UK in the early 2000s but are known to be widening. This project aimed to describe the women who died in the UK during or up to a year after the end of pregnancy, to compare the quality of care received by women from different aggregated ethnic groups, and to identify any structural or cultural biases or discrimination affecting their care.

**Methods:**

National surveillance data was used to identify all 1894 women who died during or up to a year after the end of pregnancy between 2009 and 18 in the UK. Their characteristics and causes of death were described. A Confidential Enquiry was undertaken to describe the quality of care women received. The care of a stratified random sample of 54 women who died during or up to a year after the end of pregnancy between 2009 and 18, (18 from the aggregated group of Black women, 19 from the Asian aggregated group and 17 from the White aggregated group) was re-examined specifically to describe any structural or cultural biases or discrimination identified.

**Findings:**

There were no major differences causes of death between women from different aggregated ethnic groups, with cardiovascular disease the leading cause of death in all groups. Multiple areas of bias were identified in the care women received, including lack of nuanced care (notable amongst women from Black aggregated ethnic groups who died), microaggressions (most prominent in the care of women from Asian aggregated ethnic groups who died) and clinical, social and cultural complexity (evident across all ethnic groups).

**Interpretation:**

This confidential enquiry suggests that multiple structural and other biases exist in UK maternity care. Further research on the role of microaggressions is warranted.

**Funding:**

This research is funded by the 10.13039/501100000272National Institute for Health Research (NIHR) Policy Research Programme, conducted through the Policy Research Unit in Maternal and Neonatal Health and Care, PR-PRU-1217–21,202. MK is an NIHR Senior Investigator. SK is part funded and FCS fully funded by the 10.13039/501100000272National Institute for Health Research (NIHR) Applied Research Centre (ARC) West Midlands. The views expressed are those of the author(s) and not necessarily those of the NIHR or the Department of Health and Social Care.

**Research in context**

**Evidence before this study**

Systematic review of all UK national surveillance reports describing deaths of women during pregnancy or up to a year after the end of pregnancy since 2000–2002 shows evidence of higher maternal mortality amongst women from Black and Asian aggregated ethnic groups compared to women from White ethnic groups. Similar disparities have been identified in the US and elsewhere and have been associated with racial/ethnic specific differences in the causes of death and in the quality of care women receive. There is no evidence concerning differences in factors underlying maternal deaths amongst women from different ethnic groups in the UK.

**Added value of this study**

This UK national study showed no differences in the proportionate causes of deaths during or up to a year after the end of pregnancy amongst women from different aggregated ethnic groups, nor were there any statistically significant differences in the assessed quality of care women received. Multiple areas of bias were identified in the care women received, including lack of nuanced care (notable amongst women from Black aggregated ethnic groups who died), microaggressions (most prominent in the care of women from Asian aggregated ethnic groups who died) and clinical, social and cultural complexity (evident across all ethnic groups).

**Implications of all the available evidence**

Variation in cause of death does not appear to underlie UK disparities in maternal mortality between women from different aggregated ethnic groups. Assumptions around symptomatology are made not only on the basis of pregnancy status, but also on the basis of language or ethnic group and suggest that normalisation biases may disproportionately affect women of minoritised ethnic backgrounds who die.

Alt-text: Unlabelled box

## Introduction

Racial and ethnic disparities in maternal mortality have been documented in both high^1^ and low resource settings.^2^,^3^ Disparities were first documented in the UK in the early 2000s but are known to be widening.^4^ The SARS-CoV-2 pandemic has brought them into starker focus, with infection,^5^ hospitalisation^6^ and mortality being higher amongst women from Black, Asian and other minoritised ethnic groups.

The pathways that contribute to these disparities are complex with interrelated individual, community and wider influences.^7^ They will be affected by place of birth as well as differing components of ethnicity, such as language, religion and cultural identity. In the United States (US), a substantial part of the disparity in maternal morbidity and mortality is attributed to healthcare insurance status and consequent differential access to high quality maternity care.^8^ In the UK a delayed first antenatal visit, or fewer antenatal visits than recommended, have been shown to be associated with observed differences in mortality rates between ethnic groups,^9^ but do not account for all the observed difference. Although overall the causes of maternal death in the US are similar to the UK, different causal patterns of maternal deaths amongst different racial and ethnic groups have been reported.^8^ In the UK, whilst pre-existing medical morbidities, gestational diabetes and obstetric history are known to be associated with maternal death,^9^ varying cause of death by ethnicity has not been described. Adopting the framework of Kilbourne et al,^10^ further research is therefore needed at the ‘understanding’ phase before actions can be designed to reduce the observed disparities.

Quantitative analysis of maternal mortality data alone cannot elucidate all potential factors underlying ethnic inequalities. Growing evidence from the US has shown the negative impact of ethnic and racial bias on health, both at an individual and institutional level. For example, exposure to interpersonal racial discrimination may increase the risk of poor perinatal outcome,^11^, ^12^, ^13^ and experiences of implicit or explicit racial bias within maternity care can reduce attendance at postnatal appointments.^14^ Negative experiences of care amongst women from minoritised ethnic groups have been reported in qualitative studies conducted in UK maternity settings.^15^ WHO Maternal Death Surveillance and Response (MDSR) guidance advocates maternal death review to document the frequency of both medical and non-medical contributory factors in maternal deaths, and to group the findings from maternal death reviews quantitatively to assist in prioritising responses.^16^ The aims of this project, therefore, were to describe the characteristics and causes of death of women who died in the UK during or up to a year after the end of pregnancy between 2009 and 18 and to use maternal death review to compare the quality of care received by women from different ethnic groups and explore any explicit or structural biases affecting their care.

## Methods

### Data source

MBRRACE-UK is the organisation responsible for MDSR in the UK. Surveillance and maternal death review is conducted in accordance with WHO MDSR technical guidance,^16^ and a database of all maternal deaths occurring since 2009, cross-checked with linked vital statistics records has been maintained.^17^ For the purposes of this analysis three further phases of work were undertaken in addition to the ongoing confidential enquiry:

Firstly, information on the characteristics of all women who died in the UK during or up to a year after the end of pregnancy between 2009 and 2018 was extracted from this database. Women's ethnic groups were based on maternal self-report, as detailed in their maternity records, and were classified according to the census classification for England and Wales.^18^ There are 18 groups recommended for use when asking for self-reported ethnicity, including ‘any other’ option, across five aggregated groups. For the purposes of this analysis the same terminology has been adopted, thus it refers to aggregated ethnic groups throughout. The socioeconomic characteristics of women who died were described in five aggregated groups (White, Black, Asian, Mixed and Chinese/Other). The five aggregated ethnic groups include the 18 categories used in the England and Wales census classification grouped as follows:

White: English/Welsh/Scottish/Northern Irish/British, Irish, Gypsy or Irish Traveller, Any other White background;
Black: African, Caribbean, Any other Black/African/Caribbean background;
Asian: Indian, Pakistani, Bangladeshi, Any other Asian background (Excl. Chinese);
Mixed: White and Black Caribbean, White and Black African, White and Asian, Any other Mixed/multiple ethnic background;
Chinese/Other: Chinese, Arab, Any other ethnic group not included in categories above.

### Standard confidential enquiry methodology

Each woman's cause of death was classified using the standard WHO MDSR/MBRRACE-UK methodology^16^,^17^ based on ICD-MM^19^ following review by a pathologist with expertise in maternal death autopsy with, if necessary, additional multidisciplinary discussion with a maternal death review (MDR) panel. MDR, also known as Confidential Enquiry, panels are constituted according to WHO MDSR guidance to ensure the panel has the expertise to identify both medical and non-medical problems contributing to women's deaths and for the purposes of the reviews reported here consisted of clinicians with expertise in anaesthesia, general practice, midwifery, obstetrics, obstetric medicine, pathology, psychiatry, public health, clinical epidemiology and other medical specialties, together with expertise in research into the health of women from minoritised ethnic groups, including participants from Black and other minoritised ethnic groups. MBRRACE-UK assessors are nominated after an open application process by their relevant professional organisation (for example the Royal College of Obstetricians, the Royal College of Midwives or the Obstetric Anaesthetists Association). All assessors undergo an initial training session, highlighting the principles of assessment and the guidelines for care to assess against, followed by a series of mentored assessments prior to undertaking independent assessments. Causes of death were described for women in the five aggregated ethnic groups. Public co-investigators inform the design and development of all MBRRACE-UK work but were not involved in the confidential enquiry process for confidentiality reasons. These data have not been combined or analysed in this way previously; these comparisons have not been published in MBRRACE-UK reports.

As part of the standard confidential enquiry process, each woman's care was reviewed by multidisciplinary assessors and discussed at an MBRRACE-UK MDR panel meeting, at which each woman's care was assigned an overall classification (good care, improvements in care noted which would have made no difference to outcome, improvements in care which may have made a difference to outcome). Care was assessed on a topic-specific basis for the purposes of an annual report; all causes of death are covered in a three-year cycle of reports. Therefore, the classification of care was described for women in the five aggregated ethnic groups across the three most recent published MBRRACE-UK reports (2018–20)^20^, ^21^, ^22^ to ensure that women who died from all causes were included and any differences between ethnic groups noted could not be due to differences in assessed causes of death. The proportion of women in whom ‘improvements in care were noted which may have made a difference to outcome’ were compared between each aggregated minoritised ethnic group and White women using chi squared tests. The classification of quality of care has not previously been compared across different aggregated ethnic groups. All analyses were carried out using STATA version 16 (Statacorp, TX, USA).

### Additional confidential enquiry process

Thirdly, the care of a stratified random sample of 60 women who died during or up to a year after the end of pregnancy between 2009 and 2018, (20 from the aggregated group of Black women, 20 from the Asian aggregated group and 20 from the White aggregated group) was re-examined specifically, as recommended in WHO MDSR guidance^16^,^17^ to identify evidence of the three principal mechanisms by which racism can affect health, namely structural (institutional) racism, cultural racism or discrimination^23^ in order to identify means to reduce ethnic disparities in maternal mortality. The stratified sample was drawn to include women from each aggregated ethnic group in all the regions of England and each of the four nations of the UK. A sample size of 30–50 is usually sufficient within a Confidential Enquiry to result in data saturation. We chose 20 per group (60 in total) to exceed that sample size and therefore to ensure data saturation (the point at which no new information was identified) was reached. This new assessment process was not part of the usual confidential enquiry, and for the purposes of this assessment, assessors undertook a further training session covering identification of structural and other biases and social risk factors which might impact on women's care. Women of Mixed/multiple ethnic backgrounds or Chinese/Other groups were not included in this analysis because small numbers and heterogeneity precluded inclusion of a stratified random sample. Selection was stratified based on area of residence (Northern Ireland, Scotland, Wales or region of England) as well as aggregated ethnic group. A checklist of the potential mechanisms of bias pertinent to the care of each woman was developed based on the principles of directed content analysis using existing national guidance,^24^ observed structural (institutional) racism, cultural racism or discrimination and the ‘Three Delays’ and ‘Pathway to Survival’ frameworks.^16^ Twelve experienced confidential enquiry assessors who were all part of the usual MBRRACE assessment team reviewed every woman's care and each woman's care was discussed at an MDR panel with a neutral chair. A checklist was completed for each woman by consensus panel discussion to quantify and describe potential mechanisms of bias across the three aggregated ethnic groups. Any disagreements were resolved by discussion. Checklist items are described in Table 7.

### Details of ethics approval

Identifiable MBRRACE-UK data were collected in England and Wales without consent with approval of the Secretary of State for Health and Social Care under Section 251 of the NHS Act 2006 (15/CAG/0119). Data were collected in Scotland without consent with approval from the Public Benefit and Privacy Panel for Health and Social Care (1920–0131). Identifiable information was not provided from Northern Ireland. The legal basis for this activity is Article 6 (1)(e) and Article 9 (2)(i) under the General Data Protection Regulation.

### Role of the funding source

The study sponsor (University of Oxford) played no role in study design; in the collection, analysis, and interpretation of data; in the writing of the report; or in the decision to submit the paper for publication. Marian Knight, Kathryn Bunch and Jennifer J Kurinczuk had access to the surveillance data; all authors had access to the Confidential Enquiry data. Marian Knight took the decision to submit for publication.

## Results

In total 1894 women died in the UK between 2009 and 2018 during or up to a year after the end of pregnancy, 1538 were in the White aggregated group (81%), 140 in the Black aggregated group (7%), 165 in the Asian aggregated group (9%), 21 in the Mixed aggregated group (1%) and 30 in the Chinese or Other aggregated group (2%). Thirty-nine percent of women (*n* = 741) died from direct or indirect causes during or up to six weeks after the end of pregnancy; their characteristics are shown in Table 1. Fifty-six percent of women (*n* = 1057) died between six weeks and a year after the end of pregnancy; their characteristics are shown in Table 2. The remaining five percent of women (*n* = 96) died from coincidental causes during or up to six weeks after the end of pregnancy.

**Table 1:** Characteristics of the women who died from direct or indirect causes during or up to six weeks after the end of pregnancy by aggregated ethnic group: UK 2009–2018.

	Aggregated ethnic group					
Characteristics	White N (%)	Asian N (%)	Black N (%)	Chinese/Other N (%)	Mixed N (%)	Total N (%)
**Total**	517	94	96	20	14	741
**Age (years)**						
<20	28 (5)	1 (1)	1 (1)	0 (0)	1 (7)	31 (4)
20–24	72 (14)	3 (3)	7 (7)	1 (5)	2 (14)	85 (11)
25–29	122 (24)	31 (33)	15 (16)	3 (15)	3 (21)	174 (23)
30–34	129 (25)	26 (28)	32 (33)	5 (25)	3 (21)	195 (26)
35–40	130 (25)	20 (21)	24 (25)	8 (40)	3 (21)	185 (25)
≥40	36 (7)	13 (14)	17 (18)	3 (15)	2 (14)	71 (10)
**Parity**						
0	194 (38)	31 (33)	24 (25)	5 (25)	7 (50)	261 (35)
1 to 2	217 (42)	48 (51)	51 (53)	12 (60)	6 (43)	334 (45)
≥3	87 (17)	13 (14)	15 (16)	3 (15)	1 (7)	119 (16)
Missing	19 (4)	2 (2)	6 (6)	0 (0)	0 (0)	27 (4)
**Socioeconomic status (IMD of postcode of residence)**						
First quintile (least deprived)	55 (11)	5 (5)	3 (3)	2 (10)	3 (21)	68 (9)
Second quintile	70 (14)	7 (7)	4 (4)	1 (5)	1 (7)	83 (11)
Third quintile	76 (15)	13 (14)	9 (9)	5 (25)	2 (14)	105 (14)
Fourth quintile	111 (21)	28 (30)	25 (26)	5 (25)	1 (7)	170 (23)
Fifth quintile (most deprived)	148 (29)	32 (34)	42 (44)	5 (25)	6 (43)	233 (31)
Missing	57 (11)	9 (10)	13 (14)	2 (10)	1 (7)	82 (11)
**Region of Birth**						
UK	436 (84)	27 (29)	17 (18)	4 (20)	10 (71)	494 (67)
Outside the UK	37 (7)	55 (59)	67 (70)	16 (80)	3 (21)	178 (24)
Missing	44 (9)	12 (13)	12 (13)	0 (0)	1 (7)	69 (9)
**BMI (kg/m^2^)**						
<18	6 (1)	4 (4)	1 (1)	0 (0)	0 (0)	11 (1)
18–24	173 (33)	34 (36)	24 (25)	11 (55)	4 (29)	246 (33)
25–29	104 (20)	29 (31)	26 (27)	5 (25)	2 (15)	166 (22)
≥30	154 (30)	19 (20)	31 (32)	0 (0)	7 (50)	211 (28)
Missing	80 (15)	8 (9)	14 (15)	4 (20)	1 (7)	107 (14)
**Medical or Mental Health co-morbidities**						
Yes	379 (73)	63 (67)	68 (71)	16 (80)	11 (79)	537 (72)
No	114 (22)	30 (32)	23 (24)	4 (20)	3 (21)	174 (23)
Missing	24 (5)	1 (1)	5 (5)	0 (0)	0 (0)	30 (4)

**Table 2:** Characteristics of the women who died between six weeks and one year of the end of pregnancy by aggregated ethnic group: UK 2009–2018.

	Aggregated ethnic group					
Characteristics	White inc. missing N (%)	Asian N (%)	Black N (%)	Chinese/Other N (%)	Mixed N (%)	Total N (%)
**Age (years)**						
<20	33 (3)	1 (2)	0 (0)	1 (11)	0 (0)	35 (3)
20–24	123 (13)	6 (11)	3 (8)	0 (0)	0 (0)	132 (12)
25–29	200 (21)	13 (23)	10 (28)	5 (56)	3 (50)	231 (22)
30–34	258 (27)	18 (32)	16 (44)	2 (22)	0 (0)	294 (28)
35–39	236 (25)	12 (21)	5 (14)	1 (11)	2 (33)	256 (24)
≥40	99 (10)	7 (12)	2 (6)	0 (0)	1 (17)	109 (11)
**Parity**						
0	125 (13)	15 (26)	8 (22)	4 (44)	0 (0)	152 (14)
1 to 2	249 (26)	22 (39)	17 (47)	3 (33)	3 (50)	294 (28)
≥3	111 (12)	11 (19)	7 (19)	0 (0)	2 (33)	131 (12)
Missing	464 (49)	9 (16)	4 (11)	2 (22)	1 (17)	480 (45)
**Socioeconomic status (IMD of postcode of residence)**						
First quintile (least deprived)	97 (10)	3 (5)	1 (3)	0 (0)	0 (0)	101 (10)
Second quintile	95 (10)	5 (9)	1 (3)	0 (0)	2 (33)	103 (10)
Third quintile	131 (14)	8 (14)	5(14)	5 (56)	0 (0)	149 (14)
Fourth quintile	153 (16)	11 (19)	7 (19)	2 (22)	0 (0)	173 (16)
Fifth quintile (most deprived)	233 (25)	26 (46)	14 (39)	1 (11)	3 (50)	277 (26)
Missing	240 (25)	4 (7)	8 (22)	1 (11)	1 (17)	254 (24)
**Region of Birth**						
UK	448 (47)	16 (28)	7 (19)	1 (11)	3 (50)	475 (45)
Outside the UK	51 (5)	33 (58)	26 (72)	8 (89)	0 (0)	118 (11)
Missing	450 (47)	8 (14)	3 (8)	0 (0)	3 (50)	464 (44)
**BMI (kg/m^2^)**						
<18	16 (2)	3 (5)	0 (0)	0 (0)	0 (0)	19 (2)
18–24	221 (23)	22 (39)	9 (25)	4 (44)	4 (67)	260 (25)
25–29	110 (12)	13 (23)	14 (39)	5 (56)	0 (0)	142 (13)
≥30	147 (15)	11 (19)	11 (31)	0 (0)	1 (17)	170 (16)
Missing	455 (48)	8 (14)	2 (6)	0 (0)	1 (17)	466 (44)
**Medical or Mental Health co-morbidities**						
Yes	408 (43)	34 (60)	28 (78)	3 (33)	5 (83)	478 (45)
No	129 (14)	21 (37)	6 (17)	4 (44)	1 (17)	161 (15)
Missing	412 (43)	2 (4)	2 (6)	2 (22)	0 (0)	418 (40)
						
**Total**	949	57	36	9	6	1057

The causes of death for women who died during or up to six weeks after the end of pregnancy in disease categories are shown in Table 3, and in the ICD-MM groups in Table 4. The causes of death for women who died between six weeks and a year after the end of pregnancy is shown in Table 5. There were no major differences in causes of death between women from different aggregated ethnic groups (not formally tested statistically).

**Table 3:** Cause-specific maternal deaths during pregnancy or up to six weeks after pregnancy by aggregated ethnic group: UK 2009–2018.

	Aggregated ethnic group					
Cause of Death	White N (%)	Asian N (%)	Black N (%)	Chinese/Other N (%)	Mixed N (%)	Total N (%)
**Direct deaths**						
Amniotic fluid embolism	15 (3)	5 (5)	7 (7)	*	*	27 (4)
Anaesthesia	8 (2)	*	*	*	*	8 (1)
Deaths in early pregnancy	10 (2)	*	5 (5)	*	*	19 (3)
Malignancy direct	*	*	*	*	*	*
Obstetric Haemorrhage	28 (5)	11 (12)	5 (5)	*	*	47 (6)
Pre-eclampsia and eclampsia	11 (2)	*	*	*	*	19 (3)
Suicide	31 (6)	*	*	*	*	38 (5)
Sepsis direct	22 (4)	6 (6)	6 (6)	*	*	38 (5)
Thrombosis and thromboembolism	68 (13)	9 (10)	14 (15)	*	*	93 (13)
Unascertained direct	*	*	*	*	*	*
**Indirect deaths**						
Cardiovascular disease	120 (23)	18 (19)	22 (23)	*	*	166 (22)
Malignancy indirect	9 (2)	*	*	*	*	14 (2)
Neurology	67 (13)	10 (11)	8 (8)	*	*	90 (12)
Other indirect deaths	55 (11)	13 (14)	12 (13)	*	*	82 (11)
Psychiatric non-suicide	25 (5)	*	*	*	*	26 (4)
Sepsis indirect	44 (9)	13 (14)	7 (7)	*	*	68 (9)
Influenza	21 (4)	7 (7)	*	*	*	30 (4)
Pneumonia/Others	23 (4)	6 (6)	5 (5)	*	*	38 (5)
Unascertained indirect	*)	*	*	*	*	*
**Total**	517	94	96	20	14	741

⁎ Suppressed due to small numbers (cells <5).

**Table 4:** Maternal deaths during pregnancy or in the first six weeks following using ICD-MM classification by aggregated ethnic group: UK 2009–2018.

	Aggregated ethnic group					
Cause of Death	White inc. missing N (%)	Asian N (%)	Black N (%)	Chinese/Other N (%)	Mixed N (%)	Total N (%)
**Direct Causes**						
Group 1 – Pregnancy with abortive outcome	10 (2)	3 (3)	5 (5)	0 (0)	1 (7)	19 (2)
Group 2 – Hypertensive disorders	11 (2)	3 (3)	3 (3)	1 (5)	1 (7)	19 (2)
Group 3 – Obstetric Haemorrhage	28 (5)	11 (10)	5 (5)	1 (5)	2 (13)	47 (6)
Group 4 – Pregnancy related infection	22 (4)	6 (6)	6 (6)	4 (19)	0 (0)	38 (5)
Group 5 – Other obstetric complications	116 (20)	16 (15)	23 (22)	4 (19)	2 (13)	161 (19)
Group 6 – Unanticipated complications of management	8 (1)	0 (0)	0 (0)	0 (0)	0 (0)	8 (1)
**Indirect Causes**						
Group 7 – Non-obstetric complications	322 (55)	55 (51)	54 (52)	10 (48)	8 (53)	449 (54)
Group 8 – Unknown/undetermined	0	0	0	0	0	0
**Coincidental causes**						
Group 9 – Coincidental causes	72 (12)	14 (13)	8 (8)	1 (5)	1 (7)	96 (11)
**Total**	589	108	104	21	15	837

**Table 5:** Cause-specific pregnancy-associated deaths between six weeks and one year after the end of pregnancy by aggregated ethnic group: UK 2009–2018.

	Aggregated ethnic group					
Cause of Death	White inc. missing N (%)	Asian N (%)	Black N (%)	Chinese/Other N (%)	Mixed N (%)	Total N (%)
**Direct deaths**						
Amniotic fluid embolism	*	*	*	*	*	*
Deaths in early pregnancy	*	*	*	*	*	*
Malignancy direct	*	*	*	*	*	*
Pre-eclampsia and eclampsia	*	*	*	*	*	*
Suicide	148 (16)	*	*	*	*	161 (15)
Sepsis direct	*	*	*	*	*	*
Thrombosis and thromboembolism	43 (5)	*	5 (14)	*	*	49 (5)
Total direct	200 (21)	7 (12)	6 (17)	*	*	221 (21)
**Indirect deaths**						
Cardiovascular disease	113 (12)	*	7 (19)	*	*	126 (12)
Malignancy indirect	69 (7)	5 (9)	*	*	*	76 (7)
Neurology	61 (6)	*	*	*	*	68 (6)
Other indirect deaths	72 (8)	10 (18)	*	*	*	87 (8)
Psychiatric non-suicide	114 (12)	*	*	*	*	115 (11)
Sepsis indirect	28 (3)	8 (14)	*	*	*	37 (4)
Influenza	5 (1)	*	*	*	*	7 (1)
Pneumonia/Others	23 (2)	*	*	*	*	30 (3)
Unascertained indirect	7 (1)	*	*	*	*	7 (1)
Total indirect	464 (49)	32 (56)	15 (42)	*	*	516 (49)
**Coincidental deaths**						
Malignancy coincidental	209 (22)	11 (19)	12 (33)	*	*	234 (22)
Other coincidental deaths	76 (8)	7 (12)	*	*	*	86 (8)
	285 (30)	18 (32)	15 (42)	*	*	320 (30)
**Total**	949	57	36	9	6	1057

⁎ Suppressed due to small numbers (cells <5).

The classification of the care received by the 446 women whose care was assessed as part of the Confidential Enquiry process between 2018 and 2020 is shown in Table 6. Overall, improvements in care which may have made a difference to outcome were noted for 40% (*n* = 177) women. There were no statistically significant differences in the assessed quality of care when comparing aggregated ethnic groups, noting the limited power of this national analysis to detect differences as statistically significant due to the low incidence of maternal death. Improvements in care which may have made a difference to outcome were noted for 39% of women from White aggregated ethnic groups (135/347), 48% of women from Asian aggregated ethnic groups (20/42, *p* = 0.276 compared to women from White aggregated ethnic groups), 39% of women from Black aggregated ethnic groups (15/38, *p* = 0.128 compared to women from White aggregated ethnic groups), 50% of women from Chinese/other groups (4/8, *p* = 0.525 compared to women from White aggregated ethnic groups),.and 27% of women from mixed ethnic groups (3/11, *p* = 0.435 compared to women from White aggregated ethnic groups).

**Table 6:** Classification of care received by women who died 2014–2018 in the UK whose care was reviewed as part of the Confidential Enquiry process for 2018, 2019 or 2020.

	Aggregated ethnic group					
Classification of care received	White N (%)	Asian N (%)	Black N (%)	Chinese/Other N (%)	Mixed N (%)	Total N (%)
**Good care**	118 (34)	9 (21)	13 (34)	2 (25)	6 (55)	148 (33)
**Improvements to care which would have made no difference to outcome**	94 (27)	13 (31)	10 (26)	2 (25)	2 (18)	121 (27)
**Improvements to care which may have made a difference to outcome**	135 (39)	20 (48)	15 (39)	4 (50)	3 (27)	177 (40)
P-value*	–	0.276	0.128	0.525	0.435	–
**Total**	347	42	38	8	11	446

⁎ comparing the proportion of women in whom ‘improvements in care were noted which may have made a difference to outcome’ between each aggregated minoritised ethnic group and the aggregated group of White women.

### Mechanisms of bias identified

**Figure 1:**
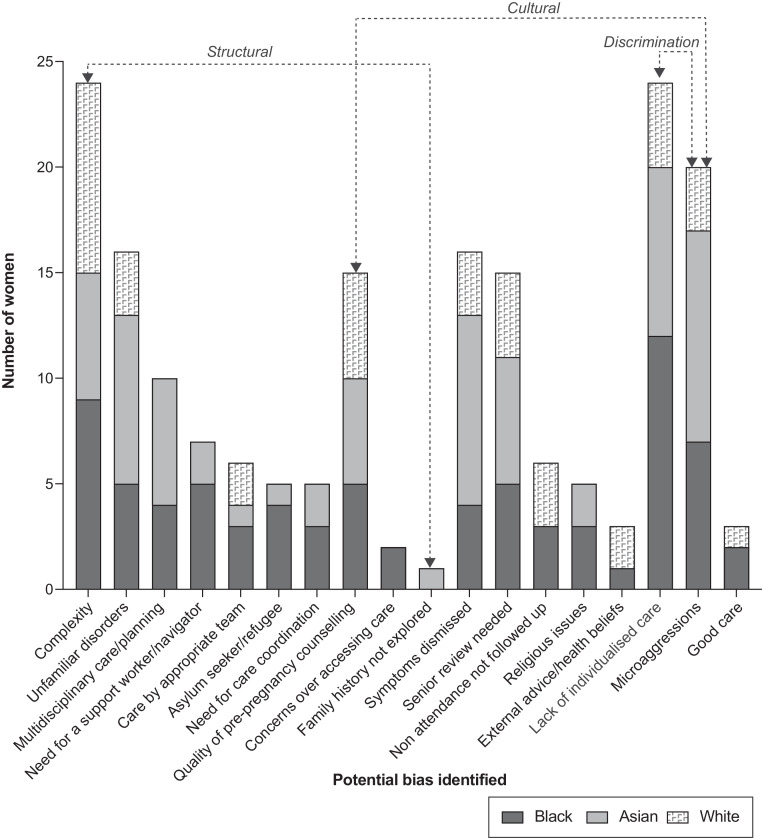
Potential biases identified in the care of women from different aggregated ethnic groups (*n* = 54 women; 18 Black, 19 Asian and 17 White).

The checklist included seventeen areas of potential structural or cultural biases or discrimination which may impact on the care received by the 60 women; specific examples of potential structural or cultural biases or discrimination identified by assessors are shown in Table 7. Assessors also noted where care had been good with no evidence of biases and efforts to provide individualised, culturally appropriate care. The numbers of women whose care was assessed to have been impacted by these potential biases is shown in Fig. 1. The care of four women (one Black, one Asian and two white) was not felt to have been impacted by any of these potential biases; three were felt to have received good care with efforts to address any potential racial or other structural biases. Multiple potential biases were identified in the care of most women (0–10 potential biases per woman who died from the Black aggregated group, median 4; 0–7 potential biases per woman who died from the Asian ethnic group, median 4; 0–6 potential biases per woman who died from the White aggregated group, median 2).

**Table 7:** Mechanisms (structural, cultural, discrimination) of potential bias identified in the care of women who died (*N* = 54).

Potential bias (mechanism)	Examples of impacts on the care of women who died
Complexity (Structural)	Current structures were unable to provide care for women with multiple complex clinical, social and cultural conditions whose needs crossed the expertise of multiple specialist teams and/or across the health/social care boundary. Structures to enable care for women with complex needs particularly needed improvement when teams were operating in different hospitals or when women relocated (or were relocated) during pregnancy.
Unfamiliar disorders (Structural)	Care for women with uncommon conditions which are frequent in specific minoritised ethnic groups was less optimal, or diagnosis was delayed in geographical areas with fewer women from minoritised ethnic groups suggesting a need to consider locations of specialist care. This was particularly evident in women with complex disease and/or atypical presentation.
Multidisciplinary care/planning (Structural)	A wider multidisciplinary team needed to be involved in women's care but this did not occur due to language barriers and/or unconscious biases.
Need for a support worker/navigator (Structural)	Women with no family/social support were not referred despite the need for a support worker. There was evidence that women were unable to attend appointments due to the complexity of navigating the system including multiple appointments in different places at different times and conflicts in scheduling between health and social care appointments.
Care by appropriate team (Structural)	Continuity of carer was infrequent, with care by an appropriate team, with appropriate expertise including safeguarding, and care in the appropriate location rarely evident.
Asylum seeker/refugee (Structural)	Restrictions due to asylum seeker/refugee status impacted on ability to receive care.
Need for care coordination (Structural)	Structural barriers such as restrictions around actions to be carried out in primary or secondary care led to a lack of continuity of therapy. This was exacerbated by lack of continuity between care provided by health and other agencies.
Quality of pre-pregnancy counselling (Structural, cultural)	Women either did not receive any pre-pregnancy counselling, or did not receive pre-pregnancy counselling, including contraception and lifestyle advice, due to language or cultural issues, indicating an need to improve both structures and cultures to enable this.
Concerns over accessing care (Structural, cultural)	Women delayed accessing care due to immigration status or not understanding UK health care entitlements indicating a need to enhance access for these vulnerable groups.
Family history not explored (Structural, cultural)	Significant family history was not explored due to language barriers or lack of awareness of its significance.
Symptoms dismissed (Cultural)	Symptoms were normalised or assumed to be due to pregnancy, by both health care professionals and women themselves, leading to lack of a proper diagnosis or underestimation of the severity of women's illness.
Senior review needed (Cultural)	Women's conditions were assumed to be caused by a disorder known to be prevalent amongst certain population groups and there was a need for senior advice which was not recognised.
Non-attendance not followed up (Cultural)	There was a lack of recognition that non-attendance/disengagement with care may be a reflection of poor experience of care and hence non-attendance was not followed up.
Religious issues (Cultural)	Religious beliefs (Christian/Muslim/Jehovah witness) impacted on concordance with care or sensitivity of care provided.
External advice/health beliefs (Cultural)	Concordance with recommended care was influenced by external advice and/or health beliefs.
Lack of individualised care (Cultural, discrimination)	Listening and learning was needed by staff together with nuance around women's background in order to provide appropriate care. Individualised care needed to move beyond consideration of solely ethnic group to include place of birth, language, cultural factors and socioeconomic background to enable appropriate care. Cognitive awareness by staff around factors such as cultural vulnerability and need for culturally appropriate lifestyle or smoking cessation advice needed improvement.
Microaggressions (Cultural, discrimination)	Multiple microaggressions suggested further need for self-awareness and cultural competency. Examples identified included: Varying descriptions of a woman's ethnic group within her records. Issues with quality of interpretation and continued reliance on interpretation by family members. Assumptions around symptomatology made on the basis of language ability and/or ethnic group. Women not listened to despite repeated presentation.
Good care	There was evidence of individualised holistic consideration of women's health needs reflecting ethnic group, language and culture socioeconomic and other health and social factors.

### Examples of biases observed

The most frequently observed biases were *“lack of individualised care”* (being treated inappropriately due to deliberate or unintentional lack of recognition of women's needs for individualised care), “*complexity*”, (multiple complex clinical, social and cultural needs which required the expertise of multiple different specialist teams) and “*microaggressions*” (defined according to Sue et al. as “brief and commonplace daily verbal, behavioural, or environmental indignities, whether intentional or unintentional, that communicate hostile, derogatory, or negative racial slights and insults toward people of colour”.^25^)

### “Lack of individualised care”

An Asian woman with difficult social circumstances and significant medical co-morbidities presented to the Emergency Department in the postnatal period with severe vomiting. Despite a significant metabolic imbalance, she was discharged with oral anti-emetics and potassium supplements. She collapsed and died a few hours later due to a cardiac arrhythmia in relation to the metabolic disturbance. Assessors felt that the woman's ethnicity meant that the extent of her social difficulties were underestimated. This influenced the decision making of the Emergency Department team and hence she was not admitted. This was a vulnerable woman for whom there was an even greater need for care and the threshold for admission should have been lower.

### “Complexity”

The woman described above illustrates some of the complex circumstances which assessors felt precluded women from receiving the care they needed. For other women, lack of health system continuity impacted the care they needed for complex medical conditions.

A woman in her forties from southern Africa, a long-term UK resident, had a complex lung condition which had necessitated a recent admission to intensive care. She was under the care of respiratory and cardiothoracic specialists but did not receive any pre-pregnancy counselling or contraceptive advice and had an unplanned pregnancy. The local referral pathways in early pregnancy excluded her general practitioner. This, in combination with a lack of comprehensive review of her medical history, which may in itself represent implicit bias, resulted in no-one in maternity services fully understanding her medical history. Although she received specialist cardiothoracic care in pregnancy, the specialist relied on the general practitioner to communicate with maternity services and this did not happen. Multiple professionals involved in her care all saw her independently and there was no multidisciplinary team assessment until she deteriorated acutely when her care was transferred to a different hospital. The maternity team were initially unaware of her transfer and there was no evidence of a documented discussion around planning mode of birth. Her condition continued to deteriorate and despite an expedited preterm caesarean birth she died.

There were several points at which continuity of specialist care could have made a difference to the outcome for this woman. Pre-pregnancy planning and contraceptive advice may have allowed her to choose not to become pregnant and at the very least would have allowed her to make an informed choice in full understanding of the possible impacts of pregnancy on her condition. It would have allowed for optimisation of her medical management before she became pregnant. Communication with her general practitioner or direct communication between respiratory, cardiothoracic and maternity teams, and comprehensive review of her medical history, would have allowed full understanding of the severity of her condition and earlier response to deterioration. Communication between hospitals would have allowed appropriate, individualised birth planning with potentially earlier intervention before she was in extremis.

### Microaggressions

These occurred in all ethnic groups but were most common in women of Asian ethnicity.

Examples amongst women from different aggregated ethnic groups included:

• Women not being listened to despite repeat presentations.
• Agitation in women who did not speak English was attributed to mental health problems when women were severely physically unwell.
• Black British women said to have a “low pain threshold”. Examples included women receiving inadequate pain relief for sickle cell crises.
• Women's ethnic group and origins described in varying ways throughout their records, from generic terms such as “Afro-Caribbean” to detailed country of birth, with varying accuracy and consistency, reflecting a lack of awareness or understanding of the importance of women's backgrounds. For example, one woman was described variously as Caribbean, from Sierra Leone and from Jamaica.

The most frequent bias impacting the care of women in the Black aggregated group was a lack of individualised care. Microaggressions, together with downgrading or dismissal of symptoms as pregnancy-related were most evident in the care of women from the Asian aggregated group. Clinical, social and cultural complexity was a key observation across all three aggregated ethnic groups; the lack of systems to provide care in the context of individual complexity was the most frequent bias identified amongst women who died from the White ethnic group.

## Discussion

Multiple potential structural biases were identified in the care women received. The most frequently observed of these were a lack of nuanced care which particularly impacted the care of women from the Black aggregated group; microaggressions, which were particularly observed in the care of women from the Asian aggregated group; and clinical, social and cultural complexity which negatively affected the care of women who died from all aggregated ethnic groups. The assessed quality of care amongst women from different aggregated ethnic groups who died during or after pregnancy was similar. Different care might have made a difference to outcome for between a third and a half of women.

This analysis used national data obtained through enhanced surveillance, and a stratified random sample for further analysis. The data therefore have a high level of completeness and are not subject to biases caused by missed deaths. Given the limited statistical power of this analysis due to small numbers of maternal deaths, despite using national data, we did not undertake comparisons using more disaggregated ethnic groups. The MDR process focussing on mechanisms of potential racial bias was conducted on a sample of women from Black, Asian and White aggregated ethnic groups, and the fact that this analysis focused on aggregated groups may be regarded as a limitation, since within each there are a wide range of different cultures, languages and religions, which may all present different influences on the care received. Current categorisation oversimplifies diversity and is likely to lead to hidden differences within groups as well as between groups. Records for a few women, principally women who died in the late postpartum period, were insufficient for assessment, and it is therefore possible that additional biases may be identifiable for this period. Whilst retrospective analysis of patient care has allowed inference about the quality of care, there may be other important differences in care that were undocumented or not sufficiently detailed in the expert reviews, such as biased attitudes or behaviours influencing non-verbal communication, and this is a limitation of solely using clinical records for assessment. It is important to note that causation cannot be inferred from this observational analysis. WHO MDSR methodology recommends the use of expert review and it should be recognised that the findings will be grounded in the opinions of the over 100 expert assessors who contributed. This research was undertaken in the context of a system of universal healthcare coverage and may not be generalisable to different healthcare systems.

Evidence of a need for nuanced, individualised care to improve women's experiences of pregnancy, labour and birth has been identified in multiple previous studies, but to our knowledge this is the first time that this has been identified so prominently amongst the care of women, particularly Black women, who died during or after pregnancy. Many previous studies have focussed on experiences of women from specific single ethnic or migrant groups, largely without significant medical or mental health co-morbidities.^26^, ^27^, ^28^ Nevertheless, a common theme described in other studies is a need to feel listened to by healthcare providers who understand their particular cultural background and needs.^29^ A national survey described the pleas of women from minoritised ethnic groups to “please believe me” and emphasises the explicit importance of feeling like an individual whose views are respected.^29^ A recent systematic review focussing specifically on experiences of perinatal mental health services in women of minoritised ethnic backgrounds highlighted particularly the impact of “insensitive and fragmented health services and interactions with culturally incompetent and dismissive health providers” on the ability of women to receive the mental health support they needed.^30^ This is an example of institutional racism, which has been described across the public sector as “The collective failure of an organisation to provide an appropriate and professional service to people because of their colour, culture, or ethnic origin. It can be seen or detected in processes, attitudes and behaviour which amount to discrimination through unwitting prejudice, ignorance, thoughtlessness and racist stereotyping which disadvantage minority ethnic people”.^31^

The framework described by Kilbourne et al^10^ divides key potential health service determinants of health inequalities into three domains: individual, provider and health care system factors. As well as individual beliefs, they recognise the importance of the consideration that health care staff (providers) may stereotype women either who hold beliefs that contrast with standard medical practice or are assumed to hold such beliefs. There is hence an important role for cultural competence in the clinical encounter to ensure the individualised care women need. Katikireddi et al., in their framework for understanding the differential impact of COVID-19 on minoritised ethnic groups^32^ emphasise, however, that many other dimensions contributing to inequality are shaped by structural racism and other power structures, including differential vulnerability to disease due, for example, to stress or living environment, differential social consequences due to working conditions (which may also affect access to care), and different effectiveness of control measures due to framing of messaging and language barriers. These latter factors reflect health care system mechanisms by which a lack of individualised care may operate to drive health inequality.

The microaggressions assessors observed in the care of women who died may also have reflected some lack of understanding by healthcare professionals of women as individuals. Racial microaggressions have been associated with delayed access to antenatal care in the US.^33^ Our study suggests microaggressions similarly exist in the UK, particularly amongst Asian women. The UK Confidential Enquiries have repeatedly noted normalisation biases underpinning maternal deaths, when concerning symptoms are repeatedly dismissed or assumed to be due to pregnancy.^20^, ^21^, ^22^^,^^34^ This analysis suggests that assumptions around symptomatology were made not only on the basis of pregnancy status, but also on the basis of language or ethnic group and suggests that normalisation biases may disproportionately affect women of minoritised ethnic backgrounds who die. Although validated instruments exist to measure microaggressions^35^ there has been little research, particularly in the UK, to assess their influence on pregnancy outcomes; our analysis suggests that further investigation is warranted.

The majority of women who die during or after pregnancy in the UK have multiple social, physical and mental health problems,^17^^,^^20^, ^21^, ^22^^,^^34^ and the impact of multiple disadvantage on women's pregnancy experiences has been highlighted previously.^36^ This analysis, however, provides evidence of structural biases – health care system factors - in UK maternity care which affect women with complex health and social problems from all ethnic groups. Current structures were unable to provide care for these particular women, whose needs required the expertise of multiple specialist teams and especially where needs traversed the health and social care boundary. This was particularly evident when teams were operating in different hospitals or when women relocated during pregnancy. Such structural biases are also hypothesised to underlie ethnic disparities in maternal morbidity and mortality in the US^8^ and represent a clear opportunity to restructure services in order to reduce disparities in maternal mortality between not only ethnic groups, but also socioeconomic groups. Kilbourne et al. describe two common features of successful interventions to reduce health disparities.^10^ Firstly, the interventions are specifically grounded within the research findings which have identified the underlying individual, provider or health system factors responsible for the observed inequality, and secondly that they were implemented using state of the art techniques amongst specific vulnerable groups. Several techniques exist to redesign services with the specific populations they serve to address their needs, including, for example, accelerated experience-based co-design using patient narratives.^37^^,^^38^ The findings described here could form a basis for a similar process.

Recent initiatives within the UK National Health Service (NHS) have been introduced in an effort to begin to tackle some of the issues identified in this analysis. The NHS Long term Plan,^39^ for example, targets continuity of midwifery carer towards women from minoritised ethnic and deprived groups, with the intention of enabling individualised and nuanced care. NHS England and NHS Improvement have developed Equity and Equality Guidance for Local Maternity Systems^40^ and local maternity services have been asked to co-produce Equity and Equality Action Plans, reflecting Kilbourne et al's observation^10^ that initiatives which are successful at reducing inequalities address the needs of specific population groups.

There was no clear evidence of differences in the quality of care offered to women who died amongst the different ethnic groups, however a constellation of biases were identified including normalisation bias, simply because women are pregnant, and structural biases limiting their ability to receive the complex and nuanced care they need. The number of biases experienced in women of minoritised ethnic backgrounds in combination with the presence of microaggressions, whether they occur because of language, culture or physical appearance, may be inferred as evidence of institutional racism. In the context of the impact of COVID-19 particularly on minoritised ethnic groups, attention to this finding is even more important and emphasises the need for continuity of pre-pregnancy, pregnancy and post-pregnancy care with someone who understands each woman as an individual, with whom she can communicate, and who is also able to navigate complex health and social care services with her. Women with complex and multiple problems need early triage to a multidisciplinary team with the requisite expertise to provide her care and healthcare structures which ensure continuity and communication can be seamlessly maintained. There is also a need to ensure the workforce is representative of the population it serves, is informed about, and able to mitigate, implicit biases and provide culturally competent care within a health system that supports them to do so. Further research on the impact of racial and other microaggressions on other pregnancy outcomes is warranted.

## Author contibutions

MK wrote the first draft of the article with contributions from KB and NV. KB and MK carried out the statistical analyses and had full access to the surveillance data. MK, KB, NV, AB, PC, FCS, MKD, JD, JG, SK, RK, RP, JS, DT, MW, JJK all conducted the Confidential Enquiry and had full access to the Confidential Enquiry data, and reviewed, edited and approved the final version of the article. MK, SK, KB and JJK contributed to the development and conduct of the study.

## Funding

This research is funded by the 10.13039/501100000272National Institute for Health Research (NIHR) Policy Research Programme, conducted through the Policy Research Unit in Maternal and Neonatal Health and Care, PR-PRU-1217–21,202. MK is an NIHR Senior Investigator. SK is part funded and FCS fully funded by the 10.13039/501100000272National Institute for Health Research (NIHR) Applied Research Centre (ARC) West Midlands. The views expressed are those of the author(s) and not necessarily those of the NIHR or the Department of Health and Social Care. All researchers were independent of the funders and all authors were not precluded from access to the data and accept responsibility for the decision to submit for publication.

## Data sharing statement

Data may be requested through the Healthcare Quality Improvement Partnership https://www.hqip.org.uk/national-programmes/accessing-ncapop-data/.

## Declaration of interest

All authors have completed the ICMJE disclosure form and declare: Marian Knight, Sara Kenyon and Jennifer Kurinczuk received grants from the NIHR PRP in relation to the submitted work. Mandish K. Dhanjal reports she is co-chair of the North West London Local Maternity System (LMS), and for the LMS, chair of the 'Reducing inequalities in maternity' subgroup. Kathryn Bunch, Nicola Vousden, Anita Banerjee, Philippa Cox, Fiona Cross-Sudworth, Jenny Douglas, Joanna Girling, Rohit Kotnis, Roshni R. Patel, Judy Shakespeare, Derek Tuffnell and Meg Wilkinson have no financial relationships with any organisations that might have an interest in the submitted work in the previous three years. No authors have other relationships or activities that could appear to have influenced the submitted work.

## Supplementary materials

Image, application 1
