# Supplementary material for: A national cohort study and confidential enquiry to investigate ethnic disparities in maternal mortality
Source: eClinicalMedicine. 2021 Dec 13;43:101237. doi: 10.1016/j.eclinm.2021.101237 (PMC8683666; doi:10.1016/j.eclinm.2021.101237)
Supplement: Supplementary file 1 [file mmc1.docx]

**Caption for supplementary material:**

Members of the MBRRACE-UK Collaboration

MBRRACE-UK Collaborators

Jennifer J Kurinczuk, Marian Knight, National Perinatal Epidemiology Unit, Nuffield Department of Population Health, University of Oxford

Elizabeth Draper, Lucy Smith, Bradley Manktelow, The Infant Mortality and Morbidity Studies (TIMMS) Team, University of Leicester

Charlotte Bevan, Janet Scott, Sands, the stillbirth and neonatal death charity:

Alan Fenton, University of Newcastle

Sara Kenyon, University of Birmingham

Rohit Kotnis General Practitioner, Oxford

Roshni Patel, Chelsea and Westminster Hospital NHS Foundation Trust, London
